# Urinary bladder hemangiosarcoma in a cat treated with partial cystectomy and adjuvant metronomic cyclophosphamide and thalidomide

**DOI:** 10.1111/jvim.16750

**Published:** 2023-06-28

**Authors:** Abigail McNally, Matteo Rossanese, Alejandro Suárez‐Bonnet, Alexandros Hardas, Andrew D. Yale

**Affiliations:** ^1^ Department of Clinical Science and Services The Royal Veterinary College, North Mymms, Hatfield Hertfordshire AL9 7TA United Kingdom; ^2^ Department of Pathobiology & Population Sciences The Royal Veterinary College, North Mymms, Hatfield Hertfordshire AL9 7TA United Kingdom; ^3^ Present address: Dick White Referrals, Station Farm, London Road, Six Mile Bottom Cambridgeshire CB8 0UH United Kingdom

**Keywords:** cat, neoplasia, oncology, veterinary

## Abstract

Visceral hemangiosarcomas (HSA) are rare in cats and typically associated with aggressive biologic behavior and poor prognosis. A 4‐year‐old male neutered domestic shorthair cat was presented with a 3‐month history of hematuria and stranguria; ultrasonography identified a large bladder mass. Complete excision was achieved by partial cystectomy. Histopathology and immunohistochemistry for von Willebrand factor confirmed HSA. The cat was treated using adjuvant cyclophosphamide, thalidomide, and meloxicam for 8 months. Abdominal ultrasonography repeated at 2 months and computed tomography repeated at 5 and 19 months after diagnosis showed no evidence of local recurrence or metastasis. The cat was alive at last follow‐up (896 days). Although the cat described in this report experienced a more favorable prognosis compared to other visceral HSA locations, additional cases are needed to further understand the biological behavior of bladder HSAs and guide treatment decisions.

**Abbreviations**
HSA: hemangiosarcoma
RI: reference interval
vWF: von Willebrand factor

## 1 INTRODUCTION

Urinary bladder neoplasia is rare in cats with an overall reported incidence of 0.07% to 0.18%, with urothelial cell carcinoma being most common.^1^, ^2^ Other reported bladder neoplasms in cats include squamous cell carcinoma, adenocarcinoma, leiomyoma, leiomyosarcoma, hemangiosarcoma, embryonal rhabdomyosarcoma, lymphoma, and angioma.^3^ Overall, hemangiosarcomas (HSA) account for <2% of all tumors in cats, with visceral forms less commonly reported compared to non‐visceral HSA. Visceral HSA in cats have been reported to involve the liver, spleen, kidney, colon, mesentery, and mediastinum.^4^, ^5^

Urinary bladder HSA has been reported very rarely in cats.^3^ It is also rare in dogs, with limited information regarding biologic behavior and clinical outcome.^6^, ^7^, ^8^, ^9^, ^10^

## 2 CASE PRESENTATION

A 4‐year‐old, 6 kg, neutered male domestic shorthair cat was referred to the oncology service of the Royal Veterinary College, United Kingdom, for evaluation of a large urinary bladder mass. Hematuria and pollakiuria first had been noted 3 months before referral. Physical examination at that time had identified a thickened bladder on palpation, but was otherwise unremarkable. Symptomatic treatment with meloxicam (0.05 mg/kg PO q24h) and amoxicillin‐clavulanate (8.75 mg/kg PO q12h) was initiated for 10 days. No improvement was seen and stranguria also developed. The dosage of amoxicillin‐clavulanate was increased to 16.75 mg/kg PO q12h for 7 more days, and the urinary supplement Cystophan (Protexin Veterinary, Somerset, United Kingdom [N‐acetyl D‐glucosamine, 125 mg; L‐tryptophan, 37.5 mg; hyaluronic acid, 10 mg]) also was initiated (2 capsules PO q24h). No further improvement was reported. An abdominal ultrasound examination was performed and identified a diffusely thickened bladder wall at the cranial pole of the bladder, with a heterogenous mass occupying the majority of the bladder lumen (3.4 × 2.5 cm). The cat was referred for further investigation.

Upon referral, physical examination identified a grade III/VI left systolic heart murmur and a large palpable bladder mass, but was otherwise unremarkable. Hematology disclosed a mildly hypochromic anemia with slight evidence of regeneration (hematocrit, 20%; reference interval [RI], 24%‐45%); red blood cell count 3.93 × 10^12^/L (RI, 5‐10 × 10^12^/L); hemoglobin 5.90 g/dL (RI 8‐15 g/dL); mean corpuscular hemoglobin concentration 29.6 g/dL (RI, 31‐35 g/dL); mean corpuscular volume 50.8 fL (RI, 39‐55 fL), and reticulocytes 0 × 10^12^/L. Serum biochemistry results were within normal limits. Because of the presence of a heart murmur, echocardiography was performed and identified hypertrophic cardiomyopathy with no evidence of left atrial enlargement.

**FIGURE 1:**
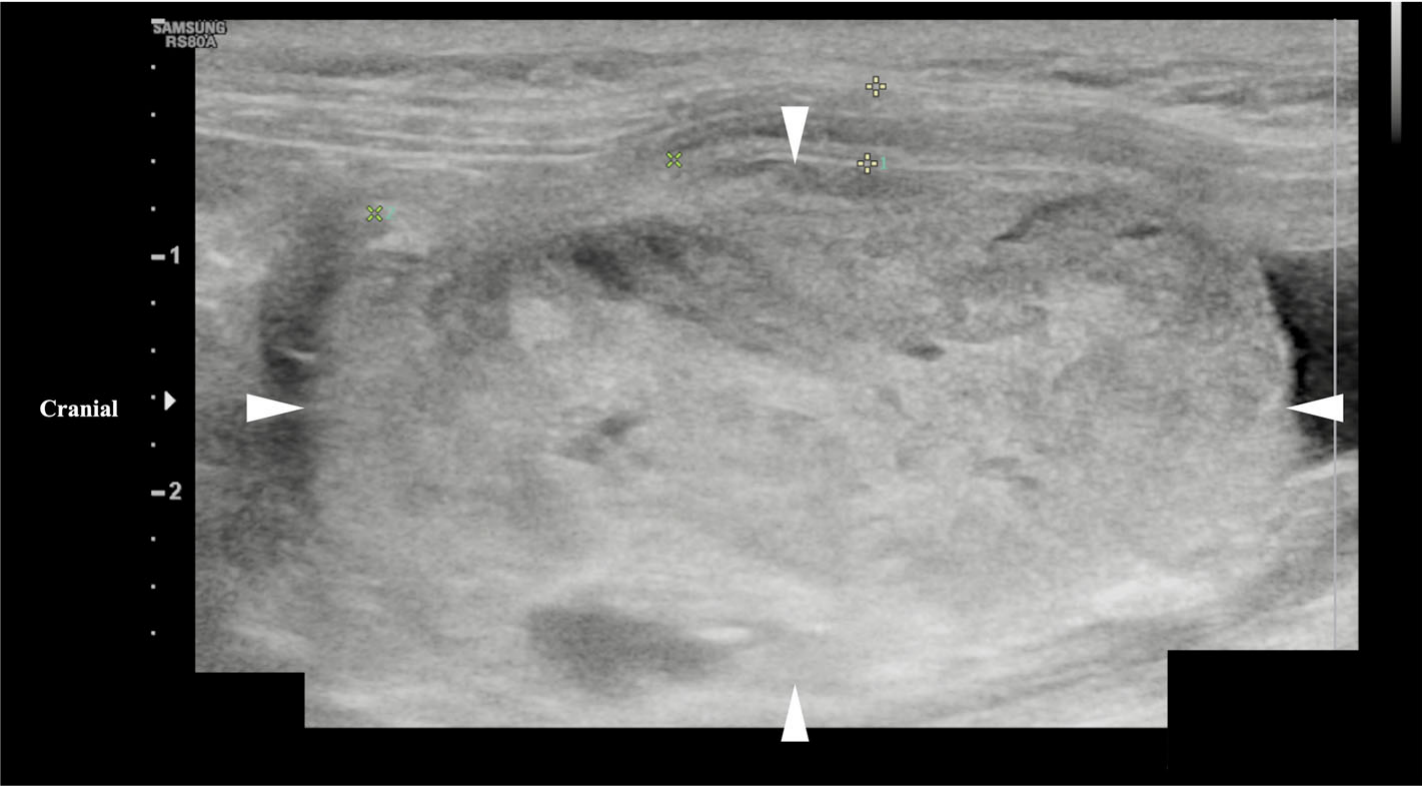
Longitudinal ultrasound image of a urinary bladder hemangiosarcoma in a cat (margins of mass highlighted by arrowheads). The image shows a smoothly marginated, heterogenous mass originating from the left cranial bladder wall and occupying approximately 80% of the bladder lumen.

Abdominal ultrasound examination identified a smoothly marginated rounded mass within the bladder, measuring 4.1 × 2.2 cm and occupying 80% of the lumen (Figure 1). It had multiple irregular anechoic regions and moderate vascular supply. At the left cranial aspect of the bladder the mass appeared confluent with the bladder wall over an area of 13 mm. This region had a focal loss of layering, and the outer margin of the bladder wall was moderately irregular. A small volume of urine was observed at the bladder neck with a moderate volume of dependent echogenic non‐shadowing sediment. A urine sample was obtained by urethral catheterization and a sample of the mass obtained by catheter suction. No other abnormalities were noted. Urine cytology identified moderate pyuria with bacteriuria and hematuria. Urine aerobic and anaerobic bacterial culture and antimicrobial sensitivity indicated a urinary tract infection with *Streptococcus lutetiensis*, sensitive to penicillin, ampicillin, and chloramphenicol. Cytology of the mass identified low numbers of epithelial cells with minimal atypia, but overall was inconclusive because of the low number of nucleated cells. Based on imaging features such as the smooth luminal surface of the mass and irregularity of the serosal margin, a smooth muscle tumor such as a leiomyoma or leiomyosarcoma was considered; other differential diagnoses included transitional cell carcinoma, rhabdomyosarcoma, fibrosarcoma or lymphoma.

**FIGURE 2:**
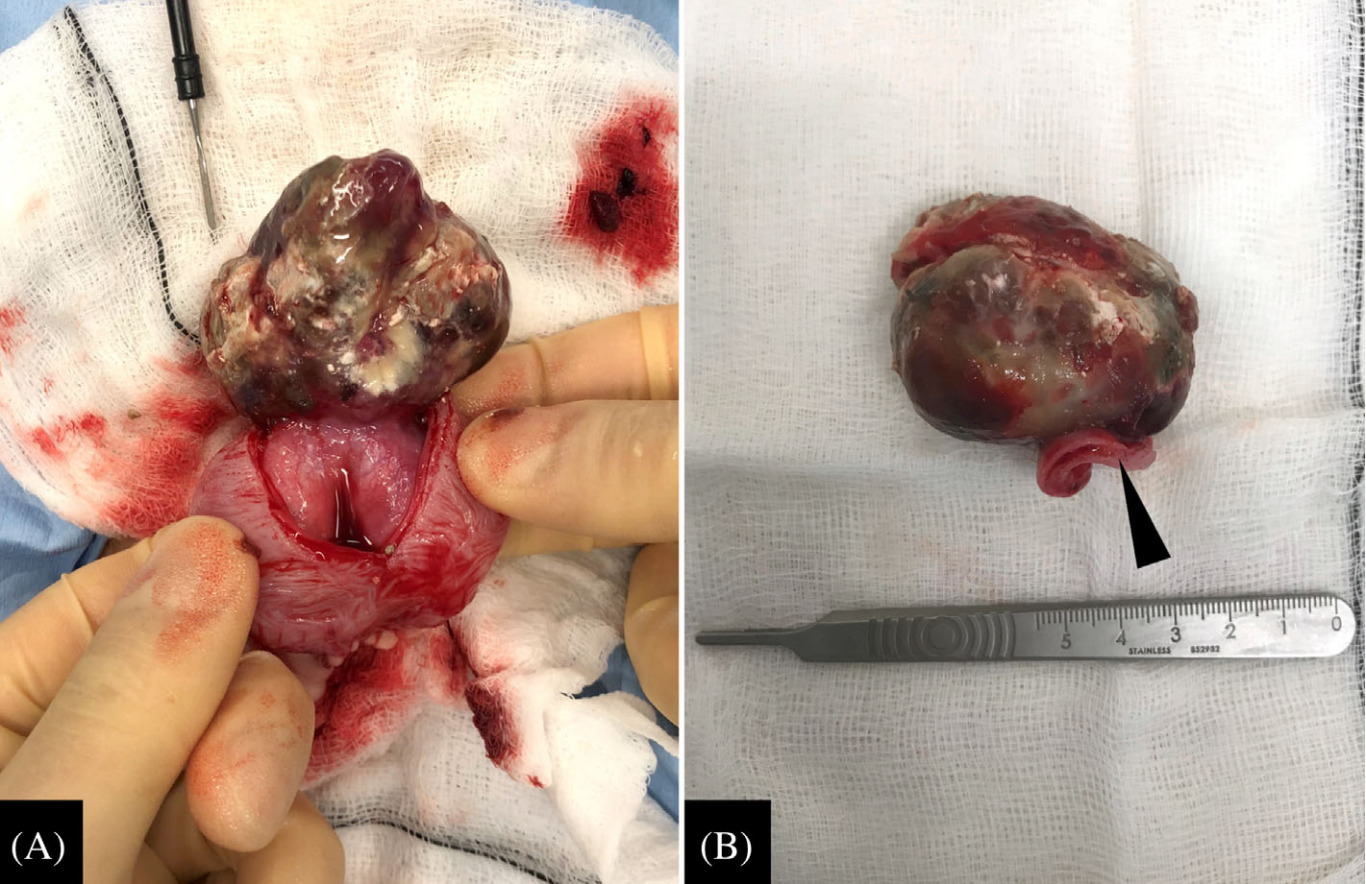
(A) Intraoperative appearance of a urinary bladder hemangiosarcoma in a cat, arising from the left craniodorsal bladder wall. (B) Gross appearance after partial cystectomy. The mass measured approximately 5 cm in length. The section of bladder wall resected with the mass is visible (arrowhead).

Because of its pedunculated morphology and location, the mass appeared amenable to surgical resection and excision by partial cystectomy was elected. A course of amoxicillin‐clavulanate (20 mg/kg PO q12h) was initiated before surgery but did not eliminate the urinary tract infection despite dose escalation. Because the mass was likely a nidus for infection, surgery was performed without resolution of the infection. At surgery a large, brown‐colored mass originating from the dorsocranial bladder wall was visualized (Figure 2). It was attached to the bladder wall with a base of approximately 1 cm and occupied the majority of the bladder lumen. The mass was excised by partial cystectomy with approximately 1 cm lateral margins. Approximately 30% of the urinary bladder was excised. The cat recovered uneventfully over the next 3 days and was discharged on amoxicillin‐clavulanate (17 mg/kg PO q12h).

**FIGURE 3:**
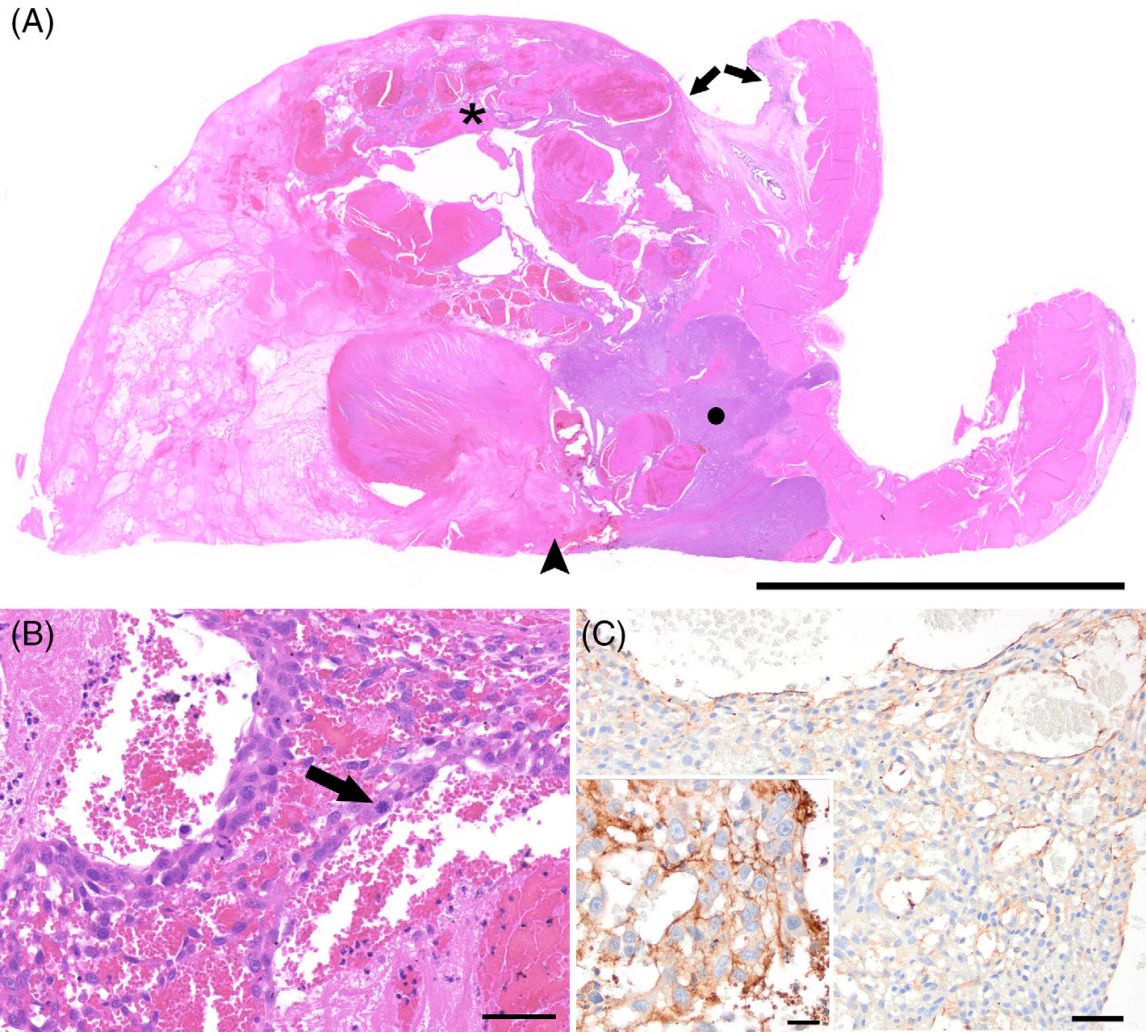
Hemangiosarcoma in the urinary bladder of a domestic shorthair cat. (A) All layers of the urinary bladder, urothelium (arrows), lamina propria, and the detrusor muscle (arrowhead) are extensively effaced multifocally and expanded by a poorly demarcated, infiltrative, malignant mesenchymal neoplasm. Neoplastic cells either form dense, confluent solid streams and whorls of plump spindloid cells (circle) or irregular vascular spaces and channels (asterisk). Hematoxylin‐eosin (HE). Bar, 1 cm. (B) Neoplastic endothelial cells have moderate anisocytosis and anisokaryosis with occasional mitoses (arrow). HE. Bar, 50 μm. (C) Neoplastic cells show positive immunolabeling of von Willebrand factor (vWF) antigen. Immunohistochemistry (IHC). Bar, 50 μm. Inset: A higher magnification view of the neoplastic endothelial cells expressing vWF antigen. IHC. Bar, 20 μm.

Histopathological examination (Figure 3) indicated that the mass extensively replaced and expanded the urothelium in a focal area. The neoplasm narrowed the lumen and expanded the lamina propria and muscularis layers of the bladder wall. It was poorly demarcated, moderately to densely cellular, exophytic, unencapsulated, infiltrative, and composed of malignant mesenchymal cells supported by a fine fibrovascular stroma. Neoplastic cells formed densely packed streams and whorls of plump spindloid cells, occasionally forming tortuous, variably sized (up to 6 mm diameter) congested blood vessels. Neoplastic cells had variably distinct cell borders, a moderate amount of eosinophilic cytoplasm, and single ovoid to elongated nuclei with 1‐2 variably distinct nucleoli. Smaller interconnected channels were lined by plump endothelial cells. Anisocytosis and anisokaryosis were moderate to marked and 11 mitoses in 10 high‐power (400×) fields (2.37 mm^2^) were observed. Within the neoplasm, approximately 70% of the section examined was affected by vascular thrombosis and coagulative necrosis. Histopathology was indicative of an HSA that was completely excised with narrow (0.7 mm) margins. The diagnosis was confirmed using immunohistochemistry for von Willebrand factor (vWF), which demonstrated positive immunolabeling of the neoplastic cells.

Adjuvant treatment using PO metronomic cyclophosphamide (15 mg/m^2^ [5 mg] PO q24h), thalidomide (1.7 mg/kg [10 mg] PO q24h) and meloxicam (0.05 mg/kg PO q24h) was initiated 18 days after surgery. Treatment was planned for a duration of 6 months. Hematology and serum biochemistry were performed 2 weeks after starting treatment, with no abnormalities reported and resolution of the previously noted anemia.

Two months after partial cystectomy, a repeat abdominal ultrasonography indicated no overt evidence of local recurrence or macroscopic regional metastasis. Further restaging, including computed tomography of the thorax and abdomen combined with focal urinary bladder ultrasonography was repeated 5 months post‐surgery. No overt evidence of regional or distant metastasis was noted. Hematology and serum biochemistry were unremarkable. Throughout this time, chemotherapy was well tolerated with no adverse effects reported. Chemotherapy was continued for an additional 4 months (8 months of treatment in total), after which further treatment was no longer possible because of owner financial limitations. Repeat staging again was performed 19 months after initial diagnosis. At this time, computed tomography of the thorax and abdomen and focal bladder ultrasonography did not identify any evidence of local recurrence or metastasis. Further restaging beyond this timepoint was declined by the owner. The cat was still alive and well at the time of last follow‐up (896 days), based on email communication with the owner.

## 3 DISCUSSION

To achieve a definitive diagnosis in this case histopathology and immunohistochemistry were required. Initial urine cytology, based on a sample collected by catheter suction, was non‐diagnostic because of low nucleated cell numbers. Previous studies indicate the detection of neoplastic cells in only 30% of urinalysis samples from dogs with lower urinary tract neoplasia, but the sensitivity and specificity of different collection methods have not been evaluated.^11^ The poor exfoliative nature of some mesenchymal tumors may have accounted for poor cellularity in the present case. Percutaneous fine needle aspiration of the mass was not considered because of the risk of needle track implantation of neoplastic cells as seen with some lower urinary tract tumors.^12^ Histologically, vascular neoplasms generally are identified by the presence of blood‐filled vascular spaces lined by neoplastic endothelial cells.^13^ In well‐differentiated vascular neoplasms diagnosis is straightforward, but in domestic species HSA diagnosis often requires additional immunohistochemistry.^13^ The main endothelial cell immunomarkers in human tissues are vWF, CD31, and CD34. However, in cats, given the broad expression of CD34 in other mesenchymal neoplasms, this marker has limited diagnostic relevance for vascular neoplasms, and vWF and CD31 are instead most reliable with 100% of hemangiomas and hemangiosarcomas in cats labeling positively for these markers in a previous study.^13^ Both vWF and CD31 also have been assessed in previous studies of dogs, with variable success in identifying neoplastic endothelial cells.^14^, ^15^ In our case, the vascular origin of the neoplastic cells was confirmed by diffuse immunohistochemical labeling of vWF.

Regarding treatment, although limited information is available to assess the utility of adjuvant chemotherapy for visceral HSA in cats, adjuvant single‐agent doxorubicin chemotherapy initially was recommended in the present case based on the aggressive nature of other visceral forms of HSA in cats and the known survival benefit of adjuvant doxorubicin‐based chemotherapy in dogs with visceral HSA.^5^, ^16^, ^17^, ^18^ However, because of the cat's temperament, injectable chemotherapy protocols were not considered feasible. Metronomic chemotherapy using cyclophosphamide, thalidomide and meloxicam instead was elected based on evidence suggesting a benefit in stage I‐II splenic HSA in dogs.^19^, ^20^ Metronomic cyclophosphamide for the treatment of HSA in cats has been described in a single case.^21^ The cat had a mid‐abdominal HSA adjacent to the mesenteric vessels and was treated with metronomic cyclophosphamide in the macroscopic disease setting. Stable disease was achieved and maintained for 8 months before the cat died because of progressive disease and hemoabdomen 10 months after diagnosis.

The cat of our report tolerated treatment with cyclophosphamide, thalidomide, and meloxicam well with no reported clinical, hematologic, or biochemical adverse effects, which is consistent with previous reports.^22^, ^23^, ^24^, ^25^ One study did not report any clinically relevant adverse effects in cats receiving metronomic cyclophosphamide‐based therapy. Only 16% and 8% of cats developed gastrointestinal and hematological adverse effects, respectively, and all were grade I‐II according to the Veterinary Cooperative Oncology Group's Common Terminology Criteria for Adverse Events (v1.1) classification scheme.^22^, ^26^ Renal toxicity is reported in 9%‐20% of cats treated with metronomic cyclophosphamide‐based therapy, but was not observed in our case.^22^, ^25^ Two studies that assessed the use of thalidomide as part of multimodal anti‐angiogenic medical treatment in cats with head and neck squamous cell carcinoma reported no adverse effects.^23^, ^24^ In these studies, a thalidomide dosage of 2 mg/kg PO q24h was used, similar to the 1.7 mg/kg PO q24h used in the present case. It is currently unclear if the efficacy of thalidomide is dose‐dependent.

No regional or distant metastases were detected in the present case, either at diagnosis or during post‐surgical follow‐up. Although the biologic behavior of bladder HSA in cats cannot be determined based on a single report, this observation could suggest a less aggressive clinical course compared to other visceral locations because, at the time of diagnosis, metastatic rates of 67% to 77% have been reported for other cats with HSA, with abdominal lymph nodes (33%) and liver (27%) representing the most common locations.^5^, ^16^, ^27^ Interestingly, in 2 clinical descriptions of urinary bladder HSA in dogs, neither case had evidence of metastasis at diagnosis.^9^, ^10^

The prognosis for bladder HSA in dogs and cats is difficult to assess given the rarity of the disease with only 5 cases previously described in dogs, and only a single histopathologic study reporting bladder HSA in a cat.^3^, ^6^, ^7^, ^8^, ^9^, ^10^ Regarding the 5 cases in dogs, 2 were reported in population studies, 1 dog was euthanized at the time of surgery, and 1 was euthanized 10 days after partial cystectomy because of bladder wall necrosis.^6^, ^7^, ^8^, ^9^ The remaining dog did not have metastasis at the time of diagnosis and was still alive 9 months after partial cystectomy, without adjuvant chemotherapy.^10^ The cat described in our study was still alive at the time of last follow‐up (896 days) with no evidence of tumor recurrence or metastasis. This outcome is longer than the previously reported median survival times for other visceral HSA in cats of between 77 and 154 days.^4^, ^5^, ^16^ The good outcome associated with bladder HSA in our case in part may be attributed to the location of the tumor, allowing for complete resection. The benefit of adjuvant chemotherapy in our case is not known.

In conclusion, we successfully treated a bladder HSA in a cat using partial cystectomy and adjuvant metronomic chemotherapy with a good long‐term outcome. Bladder HSA in cats may have a more favorable prognosis compared to other visceral locations, but review of additional cases is needed to further understand the biological behavior of bladder HSA in cats and guide treatment decisions.

## CONFLICT OF INTEREST DECLARATION

Authors declare no conflict of interest.

## OFF‐LABEL ANTIMICROBIAL DECLARATION

Authors declare no off‐label use of antimicrobials.

## INSTITUTIONAL ANIMAL CARE AND USE COMMITTEE (IACUC) OR OTHER APPROVAL DECLARATION

Authors declare no IACUC or other approval was needed.

## HUMAN ETHICS APPROVAL DECLARATION

Authors declare human ethics approval was not needed for this study.
